# The Log of the Ship's Surgeon

**Published:** 1913-09-13

**Authors:** 


### THE LOG OF THE SHIP'S SURGEON.

A recent letter, signed "Ship Surgeon," in the *Lancet* raises a point which many medical men, who some time or other in the course of their professional career have "gone down to the sea in ships," must have seriously considered on many occasions. A ship's surgeon is provided with a blank log-book. In this he is supposed to write down the details of his cases, his treatment and its results, and any observations he deems worth noting down, for the benefit of the medical superintendent of the company by which he is employed. "Ship Surgeon" states in his letter that this log-book is "open to daily critical inspection by the commander, to casual inspection by port officials at different ports, and to inspection by various people at the home port—it is also possible for various clerks in the offices to inspect it." This, we imagine, is an exceptional state of affairs, obtaining alone in the special company in which "Ship Surgeon" has been employed. Ordinarily the log-book is open to inspection by two individuals alone—the commander of the ship and the medical superintendent of the company. It is kept under lock and key on board, and although the commander has the right to read it, he seldom avails himself of that privilege; at the end of the voyage it is sent, sealed, by registered post, to the medical superintendent, and we very much doubt if that official, in any company, allows his clerks to read it. It is obvious, also, that if the surgeon wishes to protect himself from the commander's "lay criticism" he has a safeguard at hand in the use of technical terms which few sailors, we imagine, are likely to understand. While we are of opinion, therefore, that "Ship Surgeon" has somewhat overdrawn the case against the log-book, we are nevertheless with him in his argument that the doctor on board ship is not at liberty to divulge professional secrets, even though his employers may ask him to do so. The legal position on the matter is perfectly clear.

The matter is one which medical superintendents of steamship lines ought to consider attentively. All of them are capable professional men and heads of a large staff of professional men of whose obligations and privileges they are fully aware. Some, no doubt, have used antiquated forms which they found when they retired from the ship to a seat in the head office. But now that the question has been prominently raised, it is imperative that they shall make some alteration in these old-standing rules. No one wishes the abolition of the log-book; it must remain, however emasculated it may be, as a record of the duties of the surgeon, and as such is a protection both to him and to the company. But it is absurd to claim that it shall be a detailed case-book. Details of passengers' complaints are entirely unnecessary, unless they are cases of infectious disease, insanity, and accident, which, of course, must be reported to the captain. As responsible head of the vessel, in supreme authority over everyone, the surgeon included, he shares with the ship's doctor the responsibility of secrecy, and we know of no commander who is likely to divulge such secrets or to disclose the contents of the log to outsiders. It is no more a breach of professional secrecy on the part of a member of the hospital staff to report his diagnosis and treatment to the superintendent than it is for the ship's surgeon to show a similar courtesy to his commander. But it is quite a different matter to write such reports when there is a possibility that these details will come under the eye not merely of the commander of the ship, but of other and less responsible individuals. If such a possibility exists, the surgeon should refuse, even at the risk of jeopardising his position, to particularise, and should limit himself to non-committal statements, omitting his diagnosis, and writing down his treatment in the classical abbreviated style. A knowledge of the mystic signs employed by the old dispensers, and a slavish adherence to that rule, which the public still so religiously believes to be followed by the profession, that the caligraphy should be hieroglyphic to a degree bordering on absolute illegibility, will doubtless be sufficient in such cases to procure that degree of secrecy which is demanded. Nothing succeeds better in shelving an absurdity than to steep it boldly in the most respectable precedents, which are always ludicrous.

